# Neural Activation to Parental Praise Interacts With Social Context to Predict Adolescent Depressive Symptoms

**DOI:** 10.3389/fnbeh.2019.00222

**Published:** 2019-09-25

**Authors:** Stefanie L. Sequeira, Rosalind D. Butterfield, Jennifer S. Silk, Erika E. Forbes, Cecile D. Ladouceur

**Affiliations:** ^1^Department of Psychology, University of Pittsburgh, Pittsburgh, PA, United States; ^2^Department of Psychiatry, University of Pittsburgh, Pittsburgh, PA, United States

**Keywords:** social reward, adolescence, depression, peer victimization, parenting, neuroimaging (functional)

## Abstract

Negative relationships with parents and peers are considered risk factors for depression in adolescence, yet not all adolescents perceiving negative social relationships develop depression. In line with neurobiological susceptibility to social context models, we examined how individual differences in neural processing of parental praise, a unique form of social reward, might explain variability in susceptibility to perceived maternal acceptance and peer victimization. During neuroimaging, 38 11- to 17-year-olds with a history of anxiety listened to audio clips of a parent (predominately mothers) providing personalized praise and neutral statements. Average activation during parental praise clips relative to neutral clips was extracted from several anatomically-defined reward-related regions-of-interest (ROIs): the subgenual anterior cingulate cortex, caudate nucleus, amygdala, nucleus accumbens, and insula. Moderation models included direct effects and interactions between neural activation to social reward, peer victimization, and maternal acceptance at the time of scanning on depressive symptoms 1 year later. Results showed a significant three-way interaction for the bilateral caudate such that peer victimization was associated with depressive symptoms only for individuals with higher caudate response to praise who perceived maternal acceptance as low. Consistent with neurobiological susceptibility to social context models, caudate activation to social reward could represent a neural marker that helps explain variability in adolescent sensitivity to social contexts. High caudate activation to praise could reflect a history of negative experiences with parents and/or peers that places youth at greater risk for depressive symptoms. Findings suggest that interactions between neural response to reward and salient social contexts may help us understand changes in depressive symptoms during a period of development marked by significant biopsychosocial change.

## Introduction

Rates of depression increase significantly during adolescence. While only about 2%–3% of 9- to 12-year-olds meet diagnostic criteria for any depressive disorder (Costello et al., 2003), this number jumps to 10%–20% between the ages of 13 and 18 (Lewinsohn et al., 1993; Merikangas et al., 2010) and may be even higher in youth with a history of anxiety (Pine et al., 1998; Kessler et al., 2001). Research investigating biopsychosocial risk factors for major depression in early-mid adolescence (ages 9–15) suggests that negative relationships with peers and parents (Reinherz et al., 1993) and altered functioning in reward-related brain regions (Forbes and Dahl, 2012) can increase the risk for developing a depressive disorder by age 18. The joint influence of these factors has rarely been tested but may be key to understanding changes in depressive symptoms during adolescence. Developmental models suggest that social stressors influence depressive symptoms through effects on reward-related brain function (Forbes and Dahl, 2005; Nelson et al., 2005; Davey et al., 2008). A recent framework also suggests that trait-like individual differences in reward-related brain activity may help explain variability in susceptibility to negative peer and familial interactions (Schriber and Guyer, 2016). Examining how neurobiological and interpersonal factors work together to influence depressive symptoms is of heightened importance during adolescence, given significant changes in brain structure and function and reorganization of the social environment that occurs during this developmental period (Nelson et al., 2005).

Social contexts change dramatically during adolescence. The amount of time spent outside the home increases significantly from early childhood to adolescence (Gifford-Smith and Brownell, 2003), and peers begin to fulfill needs for intimacy, companionship, and reinforcement of personal worth that were previously fulfilled by parents (Rubin et al., 2006). Co-occurring with this increase in social salience of peers, however, is an increase in peer victimization. Peer victimization, also commonly labeled harassment or bullying, is common, with about 10%–20% of high school students in the US reporting moderate to high frequency of peer victimization (Nansel et al., 2001; Brunstein Klomek et al., 2007). Peer victimization in childhood and adolescence has damaging effects on psychological adjustment and is strongly associated cross-sectionally and longitudinally with symptoms of depression (Hawker and Boulton, 2000; Desjardins and Leadbeater, 2011; Ttofi et al., 2011; Stapinski et al., 2015) that can endure into adulthood (Olweus, 1993; Gladstone et al., 2006). Although the negative outcomes associated with victimization are salient and can be persistent, not all youth who experience bullying and rejection develop significant symptoms of depression. Identifying potential protective factors that make some youth more resilient to the negative effects of peer victimization is critical to developing appropriate prevention and intervention programs.

One such protective factor may be parental support and acceptance. Although adolescents become more dependent on peers during this developmental period, there is clear evidence that support from parents is still important (Colarossi and Eccles, 2003; Rueger et al., 2010). Further, greater perceived parental support and acceptance has been consistently linked to lower rates of adolescent depression (Zimmerman et al., 2000; Barber et al., 2005). The stress-buffering model proposes that high parental support and acceptance as experienced by the child can work to buffer the negative effects of peer victimization on depression (Cohen and Wills, 1985). Theoretically, peer victimization is thought to lead to a sense of incompetence and other depressive self-schemas (Bilsky et al., 2013). High perceived parental acceptance might serve as a source of positive information that can increase feelings of competence and self-worth to offset the depressive effects of peer victimization (Cole et al., 1997). Research testing the stress-buffering model has yielded mixed results, with some work finding support for this model (e.g., Bonanno and Hymel, 2010) and other work finding more support for a main effects model in which supportive parenting and peer victimization exert main effects on depressive symptoms but do not interact (e.g., Bilsky et al., 2013). Additional studies have found support for both a stress-buffering model and a main effects model (Conners-Burrow et al., 2009; Stadler et al., 2010). Most of this work relied on self-report measures of victimization, parental support, and depressive symptoms, suggesting that methodological differences likely do not entirely explain conflicting results.

An alternative explanation for the inconsistent results regarding how peer victimization and parental support influence depressive symptoms in adolescence may be individual differences in how adolescents perceive or respond to positive parenting behaviors, such as parental support and warmth. A recent framework proposed by Schriber and Guyer (2016) suggests that adolescent development is influenced by brain-based individual differences in sensitivity to social contexts, including relationships with parents and peers. The authors propose that activity in social-affective/reward-related brain regions (e.g., striatum, amygdala, insula, subgenual anterior cingulate cortex) may serve as stable, trait-like markers of sensitivity to social contexts, as these regions appear to be functionally sensitive to social experiences (for a review see Schriber and Guyer, 2016). Although brain structure and function are undoubtedly shaped by environmental influences, brain function is also largely determined by genes and is relatively stable within and across adolescence and adulthood (Manuck et al., 2007; Caceres et al., 2009; Zuo et al., 2010; Koolschijn et al., 2011). Thus, neural response to maternal praise in social reward and social-affective brain regions including the nucleus accumbens, caudate, amygdala, anterior insula, and subgenual anterior cingulate cortex, may be shaped by a combination of genes and a history of parenting influences, and this activation may provide insight into how receptive an individual is to current and future maternal warmth and acceptance.

This question is particularly relevant given that adolescence is characterized by increases in reward-seeking behavior and corresponding changes in reward-related brain circuitry (Galvan, 2010). In addition, aberrant function in regions of reward circuitry, including the striatum and medial prefrontal cortex (mPFC), has been linked to greater depressive symptoms in adolescence (for a review see Forbes and Dahl, 2012). Although early work focused on relations between depressive symptoms and neural activation to monetary rewards (e.g., Forbes et al., 2009), increasing focus is currently being paid to how alterations in neural activation to social rewards may be linked to adolescent depressive symptoms. This is especially important given that depressed mood is thought to have a strong social function (Allen and Badcock, 2003) and given the heightened salience of social-affective information during adolescence (Blakemore and Mills, 2014). Some research has found that adolescents with or at high risk for depression show attenuated neural response in the striatum and amygdala to passive social rewards, such as happy faces (Monk et al., 2008; Olino et al., 2015), as well as to active social rewards, such as maternal praise (Aupperle et al., 2016; Silk et al., 2017). However, one study found that adolescents with depression showed heightened activation to positive social feedback in subcortical structures including the amygdala (Davey et al., 2011). Depression in adolescence has also been linked to heightened amygdala activation to maternal criticism (Aupperle et al., 2016) and to heightened neural response to peer rejection in the amygdala, subgenual anterior cingulate, anterior insula, and nucleus accumbens (Silk et al., 2014). Together, these latter findings may suggest that adolescents with depression are more sensitive to social feedback, regardless of valence, in daily life.

In support of the hypothesis that social experiences may affect function in reward-related brain regions, several studies have linked normative variations in parenting behaviors to individual differences in youth’s neural responses to salient affective information from both parents and peers. For example, in a sample of 11- to 17-year-olds, Tan et al. (2014) examined how normative variations in maternal affect during a parent-child problem-solving task are associated with a child’s brain function during peer evaluation. Longer durations of maternal negative affect during the dyadic task were associated with reduced neural response to peer acceptance in the subgenual anterior cingulate, amygdala, nucleus accumbens, and anterior insula (Tan et al., 2014). This may suggest that greater maternal negative affect works to dampen the child’s neural processing of rewarding social interactions. A second study using a sample of boys also found that greater maternal warmth in early childhood (18 and 24 months), observed during mother-child interactions, was associated with reduced activation in sons’ mPFC when anticipating monetary loss in late adolescence (age 20; Morgan et al., 2014). Further, greater maternal warmth in adolescence (10 and 11 years) was associated with reduced mPFC when winning rewards and greater striatal activation when losing rewards at age 20. These findings suggest that reward-related brain regions are sensitive to maternal behaviors in childhood and adolescence. Although no work has yet been done specifically linking parental warmth and acceptance to neural activation to parental praise, Lee et al. (2014) found that perceived parental warmth was negatively correlated with neural activity in the temporoparietal junction and precuneus, social cognitive processing regions, when healthy adolescents (ages 9–17) listened to maternal criticism. The authors suggest that youth who feel more supported by their parents may be more motivated to reduce social cognitive processing while receiving criticism to protect their relationship with their parents. Together, these studies (Lee et al., 2014; Morgan et al., 2014; Tan et al., 2014) suggest potential associations between parental warmth and acceptance and brain function in adolescence.

Despite what is known about the separate effects of social stress and neural processing of social reward on depression, as well as what is known about the potential influence of social relationships on the brain, surprisingly little is known about how social stressors and brain function might interact to influence the development of depression. Developmental models posit that social stressors, including peer victimization and low parental warmth and acceptance, may influence depression through effects on neural reward circuitry (Forbes and Dahl, 2005; Nelson et al., 2005; Davey et al., 2008), though this has rarely been tested. One recent study, however, did examine specifically how low parental warmth, peer victimization, and depressive symptoms predict neural response during reward anticipation of monetary reward several years later in a large sample of adolescent girls. Casement et al. (2014) found that peer victimization in early adolescence (ages 11–12) was associated with decreased response in the mPFC to rewards in mid-adolescence (age 16). They also found that low parental warmth in early adolescence (ages 11–12) was associated in mid-adolescence (age 16) with increased activation to monetary rewards in the striatum (including the nucleus accumbens and caudate), amygdala, and the mPFC. Importantly, increased activity in the striatum and mPFC to rewards mediated the relationship between low parental warmth (ages 11–12) and depressive symptoms at age 16. Results provided initial support that normative variations in peer victimization and parental warmth may affect functioning of reward circuitry, which in turn may influence depressive symptoms. This may also suggest that high neural activity to rewards reflects past experiences of low parental warmth, which may place youth at risk for future depression. This interpretation aligns with the neurobiological susceptibility to social context framework (Schriber and Guyer, 2016). The goal of the current study was to test a complementary moderation model to further examine this framework in an independent sample, with the aim of exploring the extent to which neural activation to parental praise, a reward both social and personal in nature, may moderate the effects of concurrent perceptions of parental warmth and peer victimization on the development of depressive symptoms.

Investigating how interactions between neural reward processing and perceptions of social relationships influence depressive symptoms may be especially relevant for children with a history of anxiety, who may be at increased risk for developing depressive disorders in adolescence compared to children without a history of anxiety (Brady and Kendall, 1992; Orvaschel et al., 1995; Cummings et al., 2014). Although not all youth with anxiety will go on to develop depression, up to 75% of adolescents with depression have a history of at least one anxiety disorders (Kessler et al., 2001). Anxious youth also generally report more negative interactions with parents and peers (Ginsburg et al., 1998; Caster et al., 1999; Hale et al., 2006). Further, over 50% of youth diagnosed with anxiety disorders report being victimized by peers (Cohen and Kendall, 2015). Finally, evidence is growing for the importance of reward processing in the pathophysiology of anxiety. Research suggests that youth with anxiety and at temperamental risk for anxiety exhibit heightened neural responses to the anticipation and receipt of monetary and social rewards, especially when rewards are contingent on performance (Guyer et al., 2006, 2012; Bar-Haim et al., 2009; Benson et al., 2015). Socially anxious adolescents also exhibit heightened striatal responses to unexpected positive social feedback compared to healthy adolescents (Jarcho et al., 2015). Based on existing evidence, Silk et al. (2012) theorized that youth with anxiety disorders experience a heightened sensitivity to social evaluative threat and altered reward processing, which interact during adolescence to influence the onset of depression. Thus, interactions between neural reward processing and negative interactions with parents and peers may be particularly important for influencing depressive symptoms in youth with a history of anxiety.

## Current Study

Building off prior literature (e.g., Lee et al., 2014; Tan et al., 2014; Casement et al., 2014), and guided in part by the neurobiological susceptibility to social context framework (Schriber and Guyer, 2016), the current study aimed to address how neural activation to parental praise, perceived maternal acceptance, and peer victimization predict depressive symptoms 1 year later in adolescents ages 11–18 with a history of an anxiety disorder. Almost all parents who provided praise statements for the fMRI task were biological mothers, with the exception of one biological father. Our primary analysis tested the three-way-interaction between neural activity, maternal acceptance, and peer victimization. Aligning with the neurobiological susceptibility to social context framework (Schriber and Guyer, 2016), which suggests that youth with high neurobiological susceptibility may be more sensitive to their social contexts, we hypothesized that youth with high neural response to parental praise would show the strongest interaction between maternal acceptance and peer victimization on depressive symptoms. Specifically, we hypothesized that youth with high neural activity and low perceived maternal acceptance would show the strongest relationship between peer victimization and increases in depressive symptoms, while youth with high neural activity and high perceived maternal acceptance would show the weakest relationship between peer victimization and increases in depressive symptoms. Given the increase in depressive symptoms that occurs during mid- late-adolescence, around the same time that significant brain maturation is occurring, examining how interactions between brain function, peers, and parenting contribute to this increase may provide critical insight into how to better prevent and treat depression during this developmental period.

## Materials and Methods

### Participants

Participants were 38 youth (20 female) ranging in age from 11 to 17 years (*M*_age_ = 13.52 years, SD = 1.34). The sample was predominantly (94.7%) white. Total family income over the past year was reported on a scale of 0 (0–10,000) to 10 (100,000+). In the current sample, mean total income was between $70,000 and 80,000, with a range between $20,000 and $100,000+. See Table 1 for participant characteristics.

**Table 1:** Descriptive statistics of sample (*n* = 38).

	*n* (%)	M (SD)	Range
Age (years)	-	13.52 (1.34)	11–17
Sex–Female	20 (53%)	-	-
Total family income	-	$70,000–80,000	$20,000–100,000+
Anxiety diagnosis*
Generalized anxiety disorder	6	-	-
Social anxiety disorder	3	-	-
Separation anxiety disorder	1	-	-

Note: *Anxiety diagnoses determined at time of data collection for the current study.

Participants were recruited from a randomized control trial (RCT) to take part in the Child Anxiety Treatment Study-Depression Follow-Up (CATS-D) study. Data used for the current study were collected as part of the CATS-D study. A primary aim of CATS-D was to examine the impact of prior anxiety treatment on the development of subsequent depressive symptoms (see Silk et al., 2018). Thus, all participants had a history of an anxiety disorder. At the time of CATS-D initiation, only 9 of the 38 participants met diagnostic criteria for at least one anxiety disorder; six participants were diagnosed with generalized anxiety disorder, three were diagnosed with social anxiety disorder, and one participant was diagnosed with separation anxiety disorder. No participants had developed co-occurring MDD.

As part of the RCT from which participants were recruited, youth were randomized to 16 sessions of either cognitive behavioral therapy (CBT) or Child-Centered Therapy (CCT) at a 2:1 ratio. Full RCT procedures, including a description of diagnostic exclusionary criteria, are described in Silk et al. (2017). Briefly, exclusionary criteria included an IQ below 70 as assessed by the Wechsler Abbreviated Scale of Intelligence (Wechsler, 1997), a current primary diagnosis of major depressive disorder, attention-deficit/hyperactivity disorder (ADHD)-combined type of predominately hyperactive-impulsive type, ongoing treatment with psychoactive medication, acute suicidality or risk for harm to self or others, and failing to meet MRI safety requirements.

### Procedure

In brief, 95 participants were recruited from the RCT into CATS-D and invited to return to the lab for assessments approximately 2 years post-treatment (Time 1). All procedures were approved by a University Institutional Review Board; youth and a parent/legal guardian provided informed consent. During the first visit, clinical diagnoses were determined by a master’s level independent evaluator who was blind to treatment assignment using a semi-structured diagnostic interview. Participants completed self-report measures on depressive symptoms, perceived maternal behaviors, and peer victimization. During this visit, the participating parent, most often the biological mother, also recorded audio clips to be used in the fMRI assessment. The fMRI assessment was completed during their second visit, a few weeks following the first visit. Immediately prior to the fMRI assessment, participants were trained on the task and practiced remaining still in an MRI simulator.

As part of the CATS-D study, the self-report measure of depressive symptoms was also collected at 1-year follow-up (3 years post-treatment; Time 2). Complete data, including neuroimaging data, were available for a final sample of 38 participants. Most participating parents (*n* = 37) were biological mothers; one participating parent was a biological father. An additional nine participants had completed the neuroimaging scan but were missing data on depressive symptoms at 1-year follow-up (Time 2). These nine participants did not differ from included participants (*n* = 37) on age, sex, anxiety severity, or depressive symptoms at the time of CATS-D study initiation (all *p*s > 0.50).

### Measures

#### Structured Diagnostic Interview

The Kiddie Schedule for Affective Disorders and Schizophrenia for School-Age Children-Present and Lifetime version (K-SADS-PL; Kaufman et al., 1997), a structured diagnostic interview based on the DSM-IV (American Psychological Association, 1994), was administered by a trained clinician to all participants before confirming their inclusion in the larger study. Parents and youth were interviewed separately, with clinicians using data from both informants to arrive at a final diagnosis. Participants were included in the original treatment study if they received a diagnosis of GAD, SocAD, and/or SAD (see Silk et al., 2018). Inter-rater reliability between interviewers was calculated for 16% of interviews and found to be high (kappa = 0.97). The interview was conducted again 2-years following the completion of treatment, at the time that data collection for the current study began (Time 1). Reliability and validity analyses suggest the K-SADS-PL is a reliable and valid instrument for diagnosing anxiety disorders in children. The instrument has good test-retest reliability and high concurrent validity, such that children diagnosed with an anxiety disorder scored significantly higher than other children on self-reported anxiety measures (Kaufman et al., 1997).

#### Depressive Symptoms

The Mood and Feelings Questionnaire (MFQ; Angold and Costello, 1987) is a 33-item checklist that assesses a broad range of cognitive and vegetative symptoms of depression in children and adolescents. Each item is scored on a scale of 0 (*not true for me in the past 2 weeks*), 1 (*sometimes true for me in the past 2 weeks*), or 2 *(true for me in the past 2 weeks*), for a maximum score of 66. Children completed the child self-report version of the MFQ. In the current sample, scores on the MFQ ranged from 0 to 42 (with a mean of 9.93) at Time 1 (2 years post-treatment) and 0–30 (with a mean of 8.71) at Time 2 (3 years post-treatment). Although on average scores decreased from Time 1 to Time 2, around half the sample (*n* = 16) did show increases in depressive symptoms from Time 1 to Time 2. Scores above 27 on the MFQ may indicate the presence of depression. In the current sample, two participants had scores above 27 at Time 1 and three participants had scores above 27 at Time 2. However, no participants were diagnosed with MDD based on the K-SADS-PL. Cronbach’s alpha for the MFQ at Time 2 was 0.93.

#### Maternal Acceptance

Adolescents completed a shortened version of the Children’s Report of Parent’s Behavior Inventory (CRPBI; Schaefer, 1965; Schludermann and Schludermann, 1970). This 30-item self-report questionnaire contains descriptions of maternal child-rearing behaviors rated by children. The CRPBI includes several subscales representing three dimensions of parenting: acceptance/rejection, autonomy/psychological control, and firm/lax behavioral control. For the current study, only the acceptance/rejection dimension was used (10 items). The acceptance/rejection dimension captures the extent to which mothers express care and affection (e.g., “Tells me how much she loves me”). Children rate how much the described parenting behavior applies to their own mother using a 3-point scale from 0 = *like*, 1 = *sometimes like*, or 2 = *not like*. Cronbach’s alpha for the acceptance/rejection scale in this study was 0.89.

#### Peer Victimization

Peer victimization was measured using the Peer Relations Questionnaire (PRQ; Rigby and Slee, 1993). The PRQ is a widely-used self-report measure of bullying with three scales: a Bully scale, Victim scale, and Prosocial scale. The five-item Victim scale was used in the current study as a measure of perceived peer victimization, with scores ranging from 5 (low peer victimization) to 20 (high peer victimization). These items tap into social/relational victimization (e.g., “Other leave me out of things on purpose”), physical victimization (e.g., “I get hit and pushed around by others”), and verbal victimization (e.g., “I get called names by others”). In the current sample, scores ranged from 5 to 12. Cronbach’s alpha for the Victim scale in this study was 0.80.

#### Anxiety Symptoms

Anxiety symptoms were measured from two sources at distinct time points for use in sensitivity analyses. First, youth self-reported on their anxiety symptoms using the Screen for Child Anxiety and Related Emotional Disorders (SCARED) at Time 1 (2 years post-treatment). Cronbach’s alpha for the SCARED in this study was 0.92.

Second, as part of the larger RCT, independent evaluators rated child anxiety severity using the Pediatric Anxiety Rating Scale (PARS) at pre-treatment and post-treatment (Silk et al., 2017). A total PARS score was created by summing six items assessing anxiety severity, frequency, distress, avoidance, and interference inside and outside the home over the prior week. Treatment response was coded dichotomously; youth were considered to have responded to treatment if they demonstrated at least a 35% reduction in diagnostician-rated PARS from pre- to post-treatment (Caporino et al., 2013). Cronbach’s alpha for the PARS in this study was 0.62.

### fMRI Assessment

Participants underwent an fMRI scan during which they listened to a parent’s comments about them, delivered using MRI compatible headphones. The task included two audio clips for critical, praising, and neutral comments, which each lasted for 30 s. Procedures for obtaining the audio clips followed those used in previous studies (Hooley et al., 2005, 2009; Silk et al., 2017). Each parent produced two 30 s clips describing things that bothered her about her child (critical statements) beginning with “[Name], one thing that bothers me about you is…”, two 30 s clips describing things she likes about her child (praise statements) beginning with, “[Name], one thing I really like about you is…”, and two 30 s neutral clips about something their child would not find interesting (e.g., the weather). Critical, praising, and neutral statements were delivered in separate blocks (one block each). Each block consisted of two 30.06 s presentations (30 s audio clip and 0.06 duration to match 1.67 s TR) and three 30.06 s rest periods. The neutral block was presented first and the praise and criticism blocks were counterbalanced for order.

### BOLD Functional MRI Acquisition, Preprocessing, and Analysis

#### Imaging Acquisition

Images were acquired using a 3T Siemens Trio scanner. Blood-oxygen-level-dependent (BOLD) functional images were acquired using a T2* weighted reverse echo planar imaging (EPI) sequence. Thirty-two 3.2 mm axial slices were acquired parallel to the anterior-posterior commissure line (TR/TE = 1,670/29 ms, FOV = 205 mm, flip angle = 75°). There were three blocks. Each block lasted for 150.3 s, and 90 images were collected in each block. Before the start of the fMRI task, a high-resolution T1-weighted MPRAGE image (1 mm, axial) was collected for each participant.

#### fMRI Data Preprocessing

Images were preprocessed and analyzed using SPM12. Volumes were manually re-oriented to the anterior-posterior commissure line and corrected for slice timing. Images were then realigned to correct for motion, segmented, and co-registered to the mean functional image. Realigned images were spatially normalized to standard MNI template and smoothed with a 6 mm full-width at half-maximum Gaussian filter. Voxels were resampled during preprocessing to be 2 mm^3^. Volumes with motion greater than 5 mm/5° and global intensities more than 3 standard deviations from the mean were detected using SPM ART toolbox. Data were excluded from analyses if >25% of volumes per session were detected as outliers. Despiking was completed with interpolation using the ArtRepair toolbox in SPM. Motion parameters were included as regressors in the general linear model design in first level analyses to correct for slow-drift motion.

#### fMRI Analyses

First-level analyses included repaired pre-processed volumes, six motion parameters, and all conditions from each run (i.e., criticism, praise, neutral, rest). The contrast included for the current analyses was Praise > Neutral. Final analyses used a region-of-interest (ROI) approach. Based on similar previous literature (Silk et al., 2014, 2017; Tan et al., 2014) and based on what is known about brain regions that activate to social reward, nine *a priori* ROIs were included in current analyses—left and right nucleus accumbens, left and right caudate nucleus, left and right amygdala, left and right anterior insula, and subgenual ACC. Anatomically-defined masks for each region were created using the Talaraich atlas in the WFU PickAtlas tool (Maldjian et al., 2003). For each participant, the main effects of the task at each voxel in the brain were calculated using a *t*-statistic, producing a statistical image for each participant for the contrast of interest: Praise > Neutral. Parameter estimates for this contrast of interest were extracted from each anatomical ROI using MarsBaR (Brett et al., 2002) and loaded into SPSS v24.0.

### Data Analysis

Data were analyzed using SPSS version 24.0. All independent variables were mean-centered prior to analyses, with the exception of sex which was dummy coded (0 = male; 1 = female). Main and interactive effects of peer victimization, maternal acceptance, and neural activation to parental praise at Time 1 (2 years post-treatment) on depressive symptoms at Time 2 (1 year later; 3 years post-treatment) were examined using hierarchical linear regression. Peer victimization, maternal acceptance, neural activation to parental praise (parameter estimates), and covariates (age, sex, depressive symptoms at Time 1) were entered in Step 1. All possible two-way interactions were entered in Step 2, and the three-way interaction between peer victimization, maternal acceptance, and neural activation to parental praise was entered in Step 3. Given that peer victimization is most commonly associated with depressive symptoms, we specified the models such that peer victimization was the independent variable, with maternal acceptance and neural activation to praise as the moderators. Probing of the three-way interaction was conducted using the PROCESS macro for SPSS, version 3.1 (Hayes, 2018), which allows all study variables and covariates to be entered simultaneously and provides confidence intervals with bootstrapped standard errors (10,000 resamples).

PROCESS generates a regression model with simple slope effects. Significant interactions were probed in two ways using PROCESS: (1) examining Johnson-Neyman regions of significance (Bauer and Curran, 2005), which identifies the range of values of the moderator (in this case, neural activation) for which the association between the two-way interaction (peer victimization × maternal acceptance) and outcome (depressive symptoms) is significant; and (2) examining simple slopes of peer victimization predicting depressive symptoms at the mean and 1 SD above and below the mean of each moderator. Region of significance values were expressed in standard deviation units (mean = 0) and raw scores for ease of interpretability. Age, sex, and depressive symptoms at baseline (Time 1) were included as covariates in all analyses.

Separate models were run with parameter estimates for each ROI (nine models in total). Benjamini–Hochberg procedures (Benjamini and Hochberg, 1995) were used to account for multiple tests with a false discovery rate of 0.05.

### Sensitivity Analyses

Given that this sample received psychological treatment for an anxiety disorder, we conducted a set of sensitivity analyses to examine how treatment and/or anxiety status might impact the effects of interactions between peer victimization, maternal acceptance, and neural activation to praise on depressive symptoms. The following covariates were entered into the PROCESS macro following identification of significant models from the primary analysis: treatment type (CBT/CCT) when enrolled in the RCT, treatment response (yes/no) when enrolled in the RCT, and presence of an anxiety disorder diagnosis (yes/no) at Time 1 (2 years post-treatment). In separate models, we substituted a continuous measure of anxiety symptoms at Time 1 (SCARED scores) for the presence of an anxiety disorder diagnosis.

## Results

### Preliminary Results

Intercorrelations between variables included in the model can be found in Table 2. No marked skewness or kurtosis was found. Males and females differed significantly in perceived maternal acceptance (*t*_(36,1)_ = 3.11, *p* = 0.004), such that males reported higher acceptance than females. Males and females also differed in perceived peer victimization (*t*_(36,1)_ = −2.09, *p* = 0.044), such that females reported more peer victimization than males. Moderate correlations between age and activation in several brain regions were also found. Analyses remained controlling for sex and age. No differences between youth diagnosed with an anxiety disorder at the time of data collection vs. youth not diagnosed with an anxiety disorder were found for age, sex, depressive symptoms, maternal acceptance, peer victimization, or neural activation to parental praise in any brain region.

**Table 2:** Intercorrelations between included variables in primary analysis.

	1	2	3	4	5	6	7	8	9	10	11	12	13	14	15
1. MFQ (T1)	1														
2. MFQ (T2)	0.59***	1													
3. Age	0.28	0.16	1												
4. Sex	0.29	0.27	−0.09	1											
5. L caud	0.19	0.08	0.21	−0.04	1										
6. R caud	0.21	0.07	0.20	0.00	0.92***	1									
7. L amyg	0.08	0.19	0.18	−0.04	0.51**	0.48**	1								
8. R amyg	0.35*	0.26	0.24	−0.06	0.45**	0.44**	0.64***	1							
9. L NAcc	0.27	0.10	0.11	0.02	0.40*	0.43**	0.45**	0.57***	1						
10. R NAcc	0.22	0.08	0.17	0.12	0.46**	0.53**	0.16	0.31	0.41*	1					
11. L insula	−0.22	−0.14	0.21	0.02	0.61***	0.59***	0.55***	0.41*	0.42**	0.27	1				
12. R insula	−0.18	−0.23	0.15	−0.15	0.48**	0.54***	0.47**	0.43**	0.48**	0.20	0.85***	1			
13. sgACC	0.21	0.22	0.06	0.12	0.64***	0.65***	0.53**	0.16	0.24	0.31	0.41*	0.32	1		
14. Accept	−0.61***	−0.69***	−0.20	−0.46**	−0.19	−0.18	−0.19	−0.34*	−0.28	−0.25	0.00	0.07	−0.19	1	
15. Victim	0.23	0.26	0.04	0.33*	−0.14	−0.11	−0.03	−0.02	0.02	0.07	−0.13	0.00	−0.18	−0.24	1
															
M	9.93	8.71	13.59	0.56	0.25	0.25	−0.16	−0.06	0.03	0.08	0.01	−0.09	0.42	25.08	6.62
SD	9.57	10.13	1.51	0.50	1.14	1.04	1.50	1.55	1.56	1.34	1.38	1.13	1.27	4.37	1.99
Range	0–42	0–30	11–16	-	−4.11–2.39	−2.76–2.42	−3.38–2.55	−3.08–3.20	−2.87–2.35	−2.56–2.98	−3.06–3.18	−2.29–2.40	−3.21–2.74	17–30	5–12

Note. MFQ, Mood and Feelings Questionnaire (measure of depressive symptoms); Caud, caudate nucleus; Amyg, amygdala; NAcc, nucleus accumbens; sgACC, subgenual anterior cingulate cortex; Accept, maternal acceptance; Victim, peer victimization; M, Mean; SD, Standard Deviation; Sex is dummy coded (1 = female); ***p < 0.001, **p < 0.01, *p < 0.05.

Intercorrelations also revealed a modest correlation between activation in the right amygdala to maternal praise and perceived maternal acceptance (*r* = −0.34, *p* = 0.036), such that adolescents with greater right amygdala activation perceived lower maternal acceptance. Moderate to high correlations also emerged between perceived maternal acceptance and depressive symptoms at time 1 (*r* = −0.61, *p* < 0.001) and depressive symptoms at time 2 (*r* = −0.69, *p* < 0.001), such that adolescents reporting higher depressive symptoms also reported lower perceived maternal acceptance.

### Regression Results

In all nine ROI models, only perceived maternal acceptance was significantly associated with depressive symptoms at Time 2 (*β*s = −0.54 to −0.60, *p*s < 0.005) when main effects and covariates were entered in Step 1 (*R*^2^ = 0.54–0.56; *p*s < 0.001).

When all possible two-way interactions were entered into the models in Step 2, a significant interaction between peer victimization and activation in the left nucleus accumbens on depressive symptoms emerged (*ΔR*^2^ = 0.13, *p* = 0.021). No other significant two-way interactions emerged in other ROI models. This two-way interaction was not interpreted, as the three-way interaction between peer victimization, maternal acceptance, and left nucleus accumbens activation to praise on depressive symptoms was also significant (*ΔR*^2^ = 0.05, *p* = 0.037), though this latter finding did not survive corrections for multiple comparisons.

Following corrections for multiple tests, significant three-way interactions between peer victimization, maternal acceptance, and neural activation to praise on depressive symptoms emerged in Step 3 for two regions, the left caudate (*ΔR*^2^ = 0.11, *p* = 0.004) and right caudate (*ΔR*^2^ = 0.10, *p* = 0.007). Full results from the regression analysis for the left and right caudate are provided in Tables s 3, 4 and results from probing of these interactions using PROCESS are described below.

**Table 3:** Summary of regression model predicting depressive symptoms at 1-year follow-up using activation values from the left caudate.

	*F*	*R*^2^	Δ*F*	Δ*R*^2^	*β*	*t*	Uncorrected *p*-value	Benjamini–Hochberg *p*-value
**Model 1**	6.06***	0.54						
Age					−0.03	−0.27	0.792	
Sex					−0.11	−0.76	0.456
MFQ T1					0.27	1.74	0.091	
L Caud					−0.06	−0.50	0.620	
Peer					0.09	0.66	0.513	
Accept					−0.56	−3.40**	0.002	
**Model 2**	4.54**	0.59	1.23	0.05				
Age					−0.06	−0.45	0.658	
Sex					−0.06	−0.41	0.683	
MFQ T1					0.31	2.00	0.055	
L Caud					−0.14	−1.06	0.300	
Peer					0.11	0.81	0.424	
Accept					−0.47	−2.66*	0.013	
Peer × Accept					−0.17	−1.30	0.204	
L Caud × Accept					0.07	0.55	0.584
L Caud × Peer					0.05	0.42	0.679	
**Model 3**	6.44^***^	0.71	10.18**	0.11				
Age					−0.05	−0.39	0.701	
Sex					−0.03	−0.22	0.826	
MFQ T1					0.53	3.50**	0.002	
L Caud					−0.05	−0.41	0.685	
Peer					0.01	0.05	0.962	
Accept					−0.29	−1.77	0.088	
Peer × Accept					−0.30	−2.50*	0.019
L Caud × Accept					0.04	0.41	0.688	
L Caud × Peer					0.26	2.16*	0.040	
L Caud × Peer × Accept					−0.30	−3.19**	0.004	0.030

Note. Peer, Peer victimization; Accept, Maternal acceptance; L Caud, Left caudate activation to praise > neutral; *p < 0.05, **p < 0.005, ***p < 0.001.

**Table 4:** Summary of regression model predicting depressive symptoms at 1-year follow-up using activation values from the right caudate.

	*F*	*R*^2^	Δ*F*	Δ*R*^2^	*β*	*t*	Uncorrected *p*-value	Benjamini–Hochberg *p*-value
**Model 1**	6.09***	0.54						
Age					−0.03	−0.26	0.797	
Sex					−0.11	−0.74	0.464	
MFQ T1					0.27	1.77	0.087	
R Caud					−0.07	−0.60	0.554	
Peer					0.09	0.66	0.514	
Accept					−0.56	−3.41**	0.002	
**Model 2**	4.72**	0.60	1.44	0.06				
Age					−0.06	−0.46	0.648	
Sex					−0.03	−0.22	0.826	
MFQ T1					0.32	2.07*	0.047	
R Caud					−0.12	−0.99	0.332	
Peer					0.13	0.95	0.349	
Accept					−0.45	−2.68*	0.012	
Peer × Accept					−0.12	−0.96	0.345	
R Caud × Accept					0.08	0.72	0.477	
R Caud × Peer					0.11	0.93	0.362	
**Model 3**	6.26***	0.70	8.62**	0.10				
Age					−0.07	−0.61	0.548	
Sex					−0.04	−0.29	0.772	
MFQ T1					0.47	3.20**	0.003	
R Caud					−0.10	−0.89	0.382	
Peer					−0.01	−0.11	0.914	
Accept					−0.40	−2.64*	0.014	
Peer × Accept					−0.24	−2.01	0.054	
R Caud × Accept					0.10	0.99	0.329	
R Caud × Peer					0.18	1.71	0.099	
R Caud × Peer × Accept					−0.25	−2.94*	0.007	0.030

Note. Peer, Peer victimization; Accept, Maternal acceptance; R Caud, Right caudate activation to praise > neutral; *p < 0.05, **p < 0.005, ***p < 0.001.

#### Left Caudate

The final model was significant (*F*_(10,27)_ = 6.44, *R*^2^ = 0.70, *p* < 0.001). In addition to a main effect of depressive symptoms at time 1 [*β* = 0.53, B = 0.51 (SE = 0.15), *t*_(1,27)_ = −3.19, *p* = 0.002, 95% CI (0.22–0.84)], a significant three-way interaction between maternal acceptance, peer victimization, and left caudate activation emerged [*β* = −0.30, B = −0.34 (SE = 0.11), uncorrected *p* = 0.004, 95% CI (−0.49 to −0.11), Benjamini–Hochberg *p* = 0.03]. The Johnson-Neyman procedure revealed that the peer victimization × maternal acceptance interaction was significantly negative for values of left caudate activation above −0.09 (0.26 SDs below the mean; 63% of the sample). The effect size of the interaction increased with increasing values of left caudate activation. The Johnson-Neyman procedure also revealed that for adolescents with very low left caudate activation to praise (below −2.94 or 3.11 SDs below the mean), a significant negative interaction between peer victimization and maternal acceptance emerged. However, this only represented 2.6% of the sample, or one participant. Findings held controlling for the presence of an anxiety disorder at the time of scanning and treatment type.

**Figure 1:**
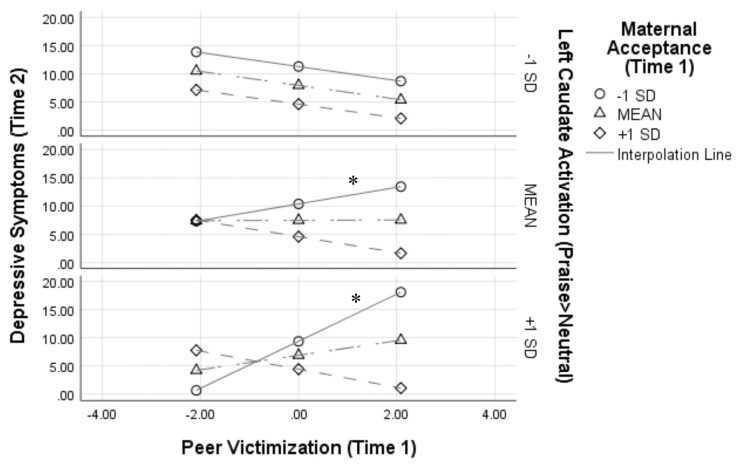
Results from the peer victimization × maternal acceptance × left caudate activation interaction on depressive symptoms 1 year later. Analyses controlled for depressive symptoms at Time 1, age, and sex. Variables were centered prior to analyses, thus a score of 0 for peer victimization represents the mean; *p < 0.05.

Figure 1 depicts this interaction by showing simple slopes representing the association between peer victimization and depressive symptoms at varying combinations of low, average, and high maternal acceptance and left caudate activation. At average and high (+1 SD) levels of left caudate activation to praise, peer victimization was positively associated with symptoms of depression only when maternal acceptance was low [simple slope at average left caudate activity: *β* = 0.30, B = 1.47 (SE = 0.71), *t*_(1,27)_ = 3.57, *p* = 0.048, 95% CI (0.003–0.60); simple slope at high left caudate activity: *β* = 0.86, B = 4.18 (SE = 1.17), *t*_(1,27)_ = 3.57, *p* = 0.013, 95% CI (0.37–1.36)].

#### Right Caudate

The final model was significant (*F*_(10,27)_ = 6.26, *R*^2^ = 0.70, *p* < 0.001). A main effect of depressive symptoms at time 1 [*β* = 0.47, B = 0.45 (SE = 0.14), *t*_(1,27)_ = 3.20, *p* = 0.004, 95% CI (0.17–0.76)] and maternal acceptance [*β* = −0.40, B = −1.13 (SE = 0.43), *t*_(1,27)_ = −2.63, *p* = 0.014, 95% CI (−0.70 to −0.08)] emerged. A significant three-way interaction between maternal acceptance, peer victimization, and left caudate activation also emerged (*β* = −0.25, B = −0.34 (SE = 0.11), uncorrected *p* = 0.007, 95% CI [−0.43 to −0.08], Benjamini–Hochberg *p* = 0.03). The Johnson-Neyman procedure revealed that the peer victimization × maternal acceptance interaction was significantly negative for values of right caudate activation above 0.16 (0.03 SDs above the mean; 45% of the sample). The effect size of the interaction increased with increasing values of right caudate activation. Findings reported held controlling for the presence of an anxiety disorder at the time of scanning and treatment type.

**Figure 2:**
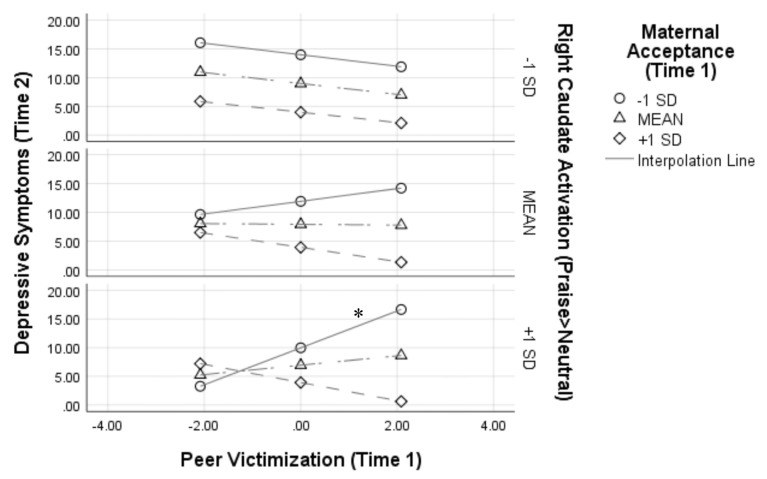
Results from the peer victimization × maternal acceptance × right caudate activation interaction on depressive symptoms 1 year later. Analyses controlled for depressive symptoms at Time 1, age, and sex. Variables were centered prior to analyses, thus a score of 0 for peer victimization represents the mean; *p < 0.05.

Figure 2 depicts this interaction by showing simple slopes representing the association between peer victimization and depressive symptoms at varying combinations of low, average, and high maternal acceptance and right caudate activation. At high (+1 SD) levels of right caudate activation to praise, peer victimization was positively associated with symptoms of depression only when maternal acceptance was low [simple slope: *β* = 0.66, B = 3.20, SE = 0.98, *t*_(1,27)_ = 3.27, *p* = 0.003, 95% CI (1.19–5.21)].

### Sensitivity Analyses

The three-way interaction between peer victimization, maternal acceptance, and left caudate activation to maternal praise on depressive symptoms remained significant when controlling for treatment type (CBT/CCT), treatment response (yes/no), and presence of an anxiety disorder diagnosis at Time 1 (2 years post-treatment; *p* = 0.022). The interaction also remained significant when controlling for treatment type, treatment response, and child-rated anxiety symptoms at Time 1 (*p* = 0.017). Similar results were seen with the right caudate. The three-way interaction between peer victimization, maternal acceptance, and right caudate activation to maternal praise on depressive symptoms remained significant when controlling for treatment type (CBT/CCT), treatment response (yes/no), and presence of an anxiety disorder diagnosis at Time 1 (2 years post-treatment; *p* = 0.045). The interaction also remained significant when controlling for treatment type, treatment response, and child-rated anxiety symptoms at Time 1 (*p* = 0.049). Treatment type, treatment response, presence of an anxiety disorder diagnosis, or child-rated anxiety symptoms were not significantly associated with depressive symptoms in any models (*p*s > 0.12).

## Discussion

The current study suggests that interactions between adolescents’ caudate activation to social reward and perceived peer victimization and maternal acceptance help explain the development of depressive symptoms 1 year later. Findings show that perceived maternal acceptance is most likely to interact with peer victimization to predict depressive symptoms for youth with higher bilateral caudate nucleus activation to parental praise. Consistent with hypotheses, youth with high caudate activation to parental praise who reported the lowest level of maternal acceptance showed the strongest positive association between peer victimization and depressive symptoms. Notably, including the three-way interaction between caudate activation, peer victimization, and maternal acceptance at Time 1 accounted for an additional 10%–11% of the variance in explaining Time 2 depressive symptoms in this sample.

Consistent with neurobiological susceptibility to social context models (Schriber and Guyer, 2016), caudate activation to social reward could represent a neural marker that helps explain variability in adolescent sensitivity to social contexts. The caudate nucleus is implicated in reward-based learning. Activity in the caudate nucleus has been positively correlated with reward prediction errors during instrumental learning tasks in both humans (O’Doherty et al., 2004; Haruno and Kawato, 2006) and monkeys (e.g., Asaad and Eskandar, 2011). Consistent with current results, Jarcho et al. (2015) recently showed that adolescents with social anxiety disorder showed significant caudate activation to unexpected positive feedback from peers of high value, corresponding to a social evaluation prediction error. One interpretation of the current findings could be that youth with positive caudate activation to praise may not have expected to hear parental praise during the task, possibly as a result of learning in the real world that positive social feedback is infrequent or fleeting. Positive caudate activation to praise could thus reflect a history of negative experiences with parents and/or peers that places youth at greater risk for depressive symptoms. This interpretation aligns with Schriber and Guyer’s (2016) proposal that neurobiological susceptibility to social context is formed throughout childhood and adolescence through ongoing consolidation of the brain’s coding of social experiences in functionally sensitive social-affective neural circuitry. This interpretation is also supported by prior work showing how parental warmth and peer victimization influences activity in reward-related brain areas (e.g., Casement et al., 2014; Morgan et al., 2014). Aligning with our interpretation that high caudate activation to praise could reflect a history of negative social experiences that places youth at risk for depressive symptoms, Casement et al. (2014) found that higher activity in a striatal region that included the caudate to reward anticipation (age 16) mediated the link between low parental warmth (ages 11–12) and higher depressive symptoms (age 16) in a sample of adolescent girls.

Current findings could also be related to the nature of this sample; that is, this is a unique sample of youth with a history of anxiety. This could help explain consistencies between current findings and prior work showing that youth with social anxiety display heightened caudate activation to unexpected positive feedback from highly valued peers compared to healthy youth (Jarcho et al., 2015). Jarcho et al. (2015) also showed that high caudate activation to unexpected positive feedback was related to disrupted recall of peer feedback. The authors suggest that social anxiety in adolescence is associated with altered neural processing of social prediction errors that contributes to impaired social learning. Results from the current study may suggest that youth with a history of anxiety demonstrating altered neural processing of social prediction errors are also most at-risk for the development of depression symptoms (Jarcho et al., 2015).

More generally, past research has also shown that youth with anxiety disorders and youth with shy/inhibited temperaments display higher caudate responses to reward than healthy youth (Guyer et al., 2006, 2012). Given the role of the caudate in motivational processes (Delgado et al., 2004), high caudate activation to social reward could reflect high approach motivation in youth with a history of anxiety (Caouette and Guyer, 2014). Although high motivation to seek out positive social experiences is likely developmentally appropriate in adolescence (Davey et al., 2008), this could lead to greater depressive symptoms when social experiences are not viewed as positive. This may be especially relevant for the current sample, as youth reporting anxiety symptoms tend to view their relationships with parents and peers as less positive (Ginsburg et al., 1998; Caster et al., 1999; Hale et al., 2006). Given that caudate activation has also been linked to various forms of arousal (e.g., Miller et al., 2014), findings may also reflect more complex influences, such as heightened fear that is characteristic of youth with anxiety (Jarcho et al., 2015). Relatedly, high caudate activity to praise may reflect greater severity of anxiety symptoms, which when combined with low parental support and high peer victimization, places adolescents at highest risk for depressive symptoms. Although current findings might not generalize to youth without a history of anxiety, this study provides valuable insight into a population of youth who are at increased risk for peer victimization and depression compared to their peers who have never been diagnosed with an anxiety disorder (Cole et al., 1998; Cohen and Kendall, 2015). Research in this population is especially important considering that over one-third of 13–18-year-olds meet criteria for an anxiety disorder (Merikangas et al., 2010). Thus, although the sample may be a limitation in that results may not generalize, it is also a unique strength.

This study benefits from the use of an ecologically-valid fMRI task and longitudinal measurement of depressive symptoms, though it has several notable limitations. First, this study relied on self-report measures of peer victimization and maternal acceptance at one point in time. Given that parental influences tend to be stronger than peer influences in childhood, with peers becoming more important into adolescence, it may be that parental tuning of reward systems early in life influences how adolescents respond not only to future parenting behaviors but also to peers. Moreover, perceptions of low parental warmth in childhood may modulate the brain’s reward system, which may impact relationships with peers. Because peer relationships are so salient in adolescence, poor relationships and increased victimization may then place adolescents at increased risk for depressive symptoms. However, future work using longitudinal measures will be needed to fully examine how the timing of peer and parental influences impacts reward-related brain development to influence depressive symptoms. This longitudinal work will also be able to address not only how perceived peer and parental interactions influence brain function, but also how brain function influences perceived social interactions. Future research could also extend beyond brain function to examine how structural brain differences, such as caudate volume, interact with an adolescent’s perceptions of social interactions to predict depressive symptoms, given evidence of altered caudate volume in adults with major depression (Krishnan et al., 1992; Kim et al., 2008).

It should also be noted that no participants in the current study were diagnosed with major depressive disorder at Time 2, and only three participants had scores on the MFQ that may indicate the presence of depression. Thus, for the majority of participants, levels of depressive symptoms at Time 2 were in a normal or subclinical range. However, good variability in depressive symptoms at Time 2 was found. We suspect that rates of depression are lower than would be anticipated in a high-risk sample due to the fact that participants previously received treatment for anxiety, which may have secondary effects on depressive symptoms (Silk et al., 2019). Additionally, results can only speak to the quality of maternal warmth, not other forms of parenting, such as harsh or inconsistent parenting. Results also cannot speak to the quality of paternal warmth, as the questionnaire was only completed about mothers. Future research assessing how other forms of parenting, child-parent attachment quality, and/or personality characteristics or psychopathology of the parent influences the associations between child brain activity, perceptions of social relationships, and depressive symptoms may be of interest. Finally, the sample was small (*n* = 38) and three-way interaction results with small sample sizes should be interpreted with caution. Interestingly, depressive symptoms did not increase with increasing levels of peer victimization for youth with low levels of caudate activation, regardless of level of maternal acceptance. Though this could suggest that low caudate activation to social reward might represent a protective marker for youth reporting high peer victimization and low maternal acceptance, this finding could be attributable to the small sample size in the current study and the small subsample with low caudate response. Though current results may be seen as preliminary, the moderate effect sizes and significant proportions of variance explained by the interactions inspire confidence that findings are meaningful. Nonetheless, future work replicating the current findings with larger samples is needed.

Findings suggest that reward-related neural circuitry may signify a biological marker of individuals who are highly susceptible to their social environments. Further, the interaction between reward-related brain function and salient social contexts may help us understand increases in depressive symptoms seen during this period of development marked by significant biopsychosocial change. This aligns with developmental psychopathology models suggesting that social stressors during childhood and adolescence can impact neural reward processing and risk for depression later in life. These results may have implications for understanding individual differences in how adolescents are affected by negative relationships with parents and peers. Further, differences in how parental support and acceptance buffer negative interactions with peers may be due, in part, to individual differences in neurobiological sensitivity to parental support and acceptance. Findings suggest that understanding increases in depressive symptoms during adolescence requires acknowledgment of both intra- and interindividual biopsychosocial factors and how these factors interact. This acknowledgment may have clinical implications for treating youth reporting significant depressive symptoms.

## Data Availability Statement

The datasets generated for this study are available on request to the corresponding author.

## Ethics Statement

This study was carried out in accordance with the recommendations of the University of Pittsburgh Institutional Review Board with written informed consent from all subjects and their primary caregiver/legal guardian. All subjects gave written informed consent in accordance with the Declaration of Helsinki. The protocol was approved by the University of Pittsburgh Institutional Review Board.

## Author Contributions

RB and SS performed the statistical analyses. JS, EF, and CL contributed to study conception and data interpretation. SS wrote the first draft of the manuscript. RB, JS, EF, and CL contributed to significant and critical revisions. All authors have read and approved the document for publication.

## Conflict of Interest

The authors declare that the research was conducted in the absence of any commercial or financial relationships that could be construed as a potential conflict of interest.
